# ENHANCING CAPACITY OF SERVICE PROFESSIONALS TO PREVENT FINANCIAL EXPLOITATION IN RURAL OLDER ADULTS WITH DEMENTIA

**DOI:** 10.1093/geroni/igad104.3304

**Published:** 2023-12-21

**Authors:** Fei Sun, Zhenmei Zhang, Ha Neul Kim, Xuehan Zhang, Yuan Xiong, Huiyu Yang, Xia Wang

**Affiliations:** Michigan State University, East Lansing, Michigan, United States; Michigan State University, East Lansing, Michigan, United States; Michigan State University, East Lansing, Michigan, United States; Michigan State University, East Lansing, Michigan, United States; Michigan State University, East Lansing, Michigan, United States; Nanjing University of Information, Science &Technology, Nanjing, Jiangsu, China (People’s Republic); Arizona State University, Phoenix, Arizona, United States

## Abstract

Cognitive impairment renders older adults more vulnerable to financial exploitation (i.e., financial abuse committed by family members, or someone known to victims, and financial fraud committed by strangers). This study developed and tested the efficacy of a community-based financial exploitation prevention model for older adults living with dementia in rural Michigan. Guided by the routine activity theory, we chose to address external risk factors for financial abuse and fraud by focusing on service professionals (i.e., social and health care providers) who are at stake for identifying and reporting abuse. Using a randomized control design, we assigned 52 service professionals in intervention groups that received training on dementia and financial exploitation and available community resources, and 39 in control groups that received community resources only. Participants were assessed at baseline and 3 months following the intervention, while 26 dropped out at 3-month follow-up. Linear mixed modeling (LMM) was used to examine time and group effects in dementia literacy, understanding of financial exploitation, and competence in handling financial exploitation. Both groups reported an increase in their knowledge of dementia and their competence of handing financial exploitation cases. Moreover, those in the intervention groups reported more understanding of risk factors for financial abuse committed by family members. Community service agencies comprise a strong safety network for vulnerable older adults at risk for financial exploitation. Policies and practice need to be in place to sustain the coordinated efforts among community service agencies and adult protective services to prevent financial exploitation.

